# Fast high-resolution metabolic imaging of acute stroke with 3D magnetic resonance spectroscopy

**DOI:** 10.1093/brain/awaa264

**Published:** 2020-11-03

**Authors:** Yao Li, Tianyao Wang, Tianxiao Zhang, Zengping Lin, Yudu Li, Rong Guo, Yibo Zhao, Ziyu Meng, Jun Liu, Xin Yu, Zhi-Pei Liang, Parashkev Nachev

**Affiliations:** a1 Institute for Medical Imaging Technology, School of Biomedical Engineering, Shanghai Jiao Tong University, Shanghai, China; a2 Radiology Department, Shanghai Fifth People’s Hospital, Fudan University, Shanghai, China; a3 Beckman Institute for Advanced Science and Technology, University of Illinois at Urbana-Champaign, Urbana, IL, USA; a4 Department of Electrical and Computer Engineering, University of Illinois at Urbana-Champaign, Urbana, IL, USA; a5 Department of Biomedical Engineering, Case Western Reserve University, Cleveland, OH, USA; a6 High-Dimensional Neurology Group, Institute of Neurology, University College London, London, UK

**Keywords:** ischaemic stroke, magnetic resonance spectroscopic imaging, lactate, *N*-acetylaspartate, penumbra

## Abstract

Impaired oxygen and cellular metabolism is a hallmark of ischaemic injury in acute stroke. Magnetic resonance spectroscopic imaging (MRSI) has long been recognized as a potentially powerful tool for non-invasive metabolic imaging. Nonetheless, long acquisition time, poor spatial resolution, and narrow coverage have limited its clinical application. Here we investigated the feasibility and potential clinical utility of rapid, high spatial resolution, near whole-brain 3D metabolic imaging based on a novel MRSI technology. In an 8-min scan, we simultaneously obtained 3D maps of *N*-acetylaspartate and lactate at a nominal spatial resolution of 2.0 × 3.0 × 3.0 mm^3^ with near whole-brain coverage from a cohort of 18 patients with acute ischaemic stroke. Serial structural and perfusion MRI was used to define detailed spatial maps of tissue-level outcomes against which high-resolution metabolic changes were evaluated. Within hypoperfused tissue, the lactate signal was higher in areas that ultimately infarcted compared with those that recovered (*P < *0.0001). Both lactate (*P < *0.0001) and *N*-acetylaspartate (*P < *0.001) differed between infarcted and other regions. Within the areas of diffusion-weighted abnormality, lactate was lower where recovery was observed compared with elsewhere (*P < *0.001). This feasibility study supports further investigation of fast high-resolution MRSI in acute stroke.

## Teaser

Li *et al*. demonstrate the feasibility of rapid, high-resolution, near whole-brain 3D MR spectroscopic imaging in acute stroke within real-world clinical environments. Metabolic maps obtained at 3 mm spatial resolution show that the lactate signal can distinguish between areas of infarction, penumbra and oligaemia.

## Introduction

High-resolution mapping of brain tissue viability is key to optimal therapeutic selection and outcome prediction in acute stroke (van Leyen *et al.*, 2019). Though viability is a functional characteristic, most closely assayed by cellular metabolism (Astrup *et al.*, 1981), current clinical practice relies on structural and vascular characteristics as a surrogate for it. MRI with diffusion- and perfusion-weighting (DWI and PWI) have been used to map ischaemic brain tissue damage, with the mismatch between DWI lesion and PWI-identified region of hypoperfusion considered as the salvageable penumbra (Schlaug *et al.*, 1999). However, the accuracy of observed mismatch is relatively poor because the perfusion deficit region often contains oligemic tissue, and the DWI lesion can reverse if treated promptly (Ringer *et al.*, 2001; Fiehler *et al.*, 2002; Kidwell *et al.*, 2003; Sobesky *et al.*, 2005; Guadagno *et al.*, 2006; Leigh *et al.*, 2018). PET can quantify cerebral metabolism; indeed it is currently the gold standard for penumbra detection, but its clinical use has been limited owing to the requirement for radioactive tracers (Zaro-Weber *et al.*, 2019).

Magnetic resonance spectroscopic imaging (MRSI) has long been recognized as a potentially powerful tool for non-invasive detection of neurometabolic alterations induced by stroke. Amongst other key metabolites, ^1^H-MRSI allows the measurement of *N*-acetylaspartate (NAA), a marker of neuronal integrity, and lactate, a marker of anaerobic glycolysis. Following the first clinical MRSI study of stroke in 1994 (Barker *et al.*, 1994), significant efforts have been made to further develop MRSI and evaluate its potential for the assessment of brain ischaemia tissue (Wild *et al.*, 2000; Nicoli *et al.*, 2003; Munoz Maniega *et al.*, 2008; Cvoro *et al.*, 2009). An important suggestive finding from these studies was that the preservation of NAA coupled with an increase in lactate may be a surrogate biomarker of the ischaemic penumbra (Gillard *et al.*, 1996; Dani and Warach, 2014). However, the long data acquisition time (over 5 min for a single slice), low spatial resolution (over 20 mm for single voxel and over 10 mm for 2D imaging), and narrow tissue coverage have significantly obstructed the translation of the technique into real-world clinical practice.

In the present study, we sought to demonstrate the feasibility and clinical potential of rapid, high-resolution, near whole-brain 3D metabolic imaging of stroke using a newly developed ^1^H-MRSI technology known as SPICE (SPectroscopic Imaging by exploiting spatiospectral CorrElation) (Liang, 2007; Lam and Liang, 2014; Lam *et al.*, 2016; Li *et al.*, 2017; Peng *et al.*, 2018; Guo *et al.*, 2019). In an 8-min scan, we simultaneously obtained 3D maps of NAA, lactate and other metabolites at a nominal spatial resolution of 2.0 × 3.0 × 3.0 mm^3^. We evaluated the tissue-level neurometabolic changes in acute stroke patients and explored its relationship with final tissue outcome. In combination with established DWI and PWI methods, we also explored whether high-resolution 3D metabolic mapping can improve the delineation of ischaemic penumbra without any exogenous label.

## Materials and methods

### Patients

We prospectively recruited 31 consecutive ischaemic stroke patients within 24 h of symptom onset, regardless of age or stroke severity. This study was approved by the Institutional Review Board of Shanghai Fifth People’s Hospital, China. Written informed consent was obtained from all participants. Exclusion criteria included the presence of a contraindication for MRI, haemorrhage, or a non-stroke lesion on structural MRI. The patients were otherwise unselected. All clinical decisions such as administration of thrombolysis were made prior to enrolment to avoid introducing any delay to best clinical care. Some of the scanning was performed while thrombolysis was taking place.

Of the 31 eligible patients who received an initial MRI scan, 13 were excluded from final data analysis (one with haemorrhagic stroke; two with pre-existing lesions; three with no visible DWI lesion; two owing to motion corruption; three owing to artefacts in the MRSI images caused by residual water and lipid signals; two were unable to complete the scan). There was otherwise no missing data. Therefore, 18 patients were included in the analysis, with a mean age of 67 years (range 40–84 years, SD 13 years). The median National Institute for Health Stroke Scale was 6 (range 1–12). Two patients received thrombolytic therapy. The characteristics of the included patients are summarized in Supplementary Table 1.

### MRI

**Figure 1:**
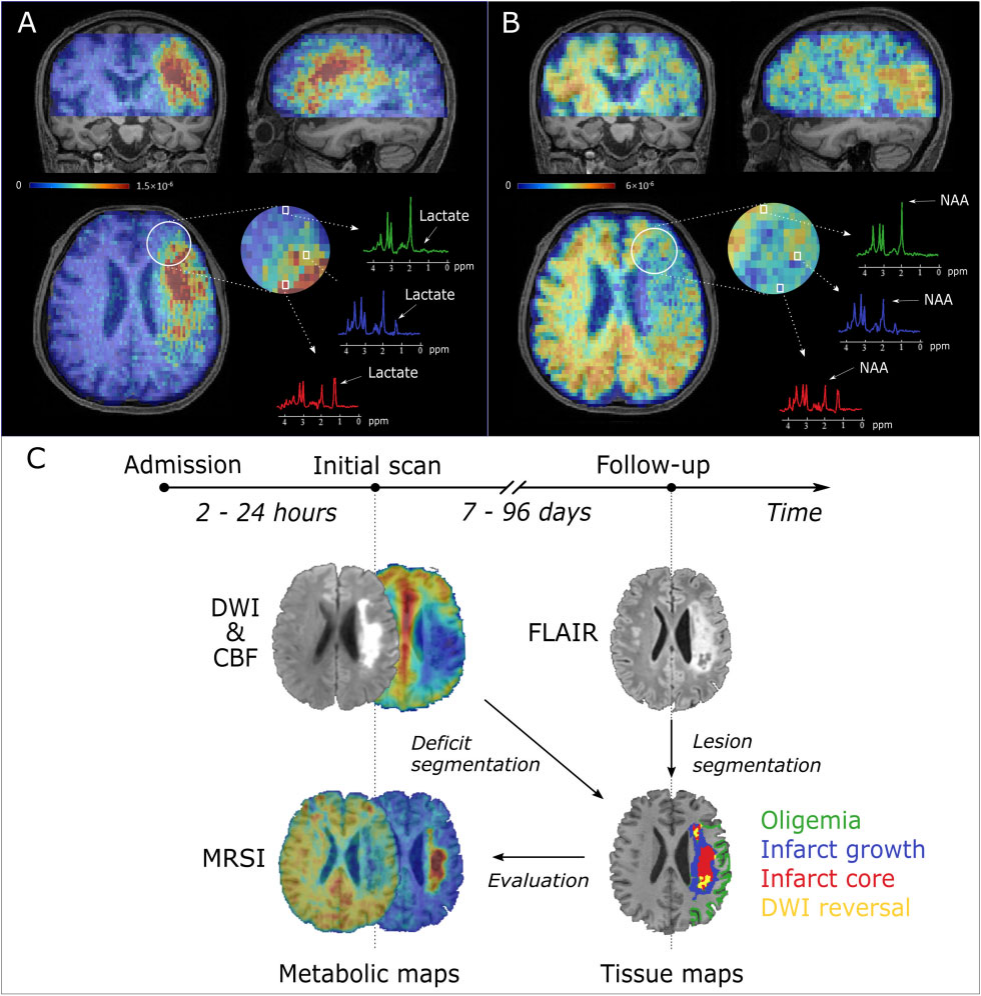
**3D MRSI for an acute ischaemic stroke patient brain at a nominal spatial resolution of 2.0 × 3.0 × 3.0 mm^3^ in an 8-min scan.** (**A**) 3D lactate map in triplanar views overlaid on T_1_-weighted images. The representative spectra were acquired from the infarct core (red), infarct growth (blue) and oligemia (light green) regions, respectively. (**B**) 3D NAA map in triplanar views overlaid on T_1_-weighted images. The representative spectra were acquired from the infarct core (red), infarct growth (blue) and oligemia (light green) regions, respectively. (**C**) Timeline of the experimental study.

All scans were performed using a 3T Siemens Skyra MR scanner. The initial MRI and MRSI images were acquired upon patients’ admission, with the time from symptom onset to scan at 2 to 24 h. The follow-up MRI scanning was repeated at 7–96 days to determine the size of the infarct. The timeline of imaging is shown in Fig. 1C. The sequences for initial MRI scans included DWI (1.3 × 1.3 × 4.0 mm^3^, field of view = 240 mm, *b *=* *0 and *b *=* *1000 s/mm^2^, repetition time = 5200 ms, echo time = 64 ms, 25 slices) for apparent diffusion coefficient (ADC) calculation and 3D MPRAGE imaging (1.0 × 1.0 × 1.0 mm^3^, field of view = 256 mm, repetition time = 2400 ms, echo time = 2.13 ms, inversion time = 1100 ms, 192 slices). The follow-up scans included FLAIR imaging (0.5 × 0.5 × 2.0 mm^3^, field of view = 240 mm, repetition time = 9000 ms, echo time = 89 ms, 82 slices).

### Magnetic resonance spectroscopic imaging

High-resolution 3D metabolic imaging was performed in the initial scan using the latest SPICE ^1^H-MRSI sequence that covered a field of view of 240 × 240 × 72 mm^3^ with 2.0 × 3.0 × 3.0 mm^3^ nominal spatial resolution in 8 min (Peng *et al.*, 2018; Guo *et al.*, 2019). This was made possible using several novel data acquisition and processing features: (i) elimination of both water and lipid suppression that are commonly used in conventional MRSI data acquisition methods; (ii) use of free induction decays instead of spin echoes to encode spatiospectral information; (iii) use of an ultrashort echo time (1.6 ms) and short repetition time (160 ms); (iv) sparse sampling of (k, t)-space; (v) acquisition of navigators for tracking magnetic field drift and subject motion; and (vi) use of advanced data processing methods for effective removal of residual water and lipid signals and for data denoising. The ultrashort echo time acquisitions also improved signal-to-noise ratio while short repetition time and sparse sampling of (k, t)-space enabled rapid data acquisition.

The spatiospectral function containing the desired neurometabolic information was reconstructed from the MRSI data collected with the SPICE sequence using a union-of-subspaces model, incorporating pre-learned spectral basis functions (Liang, 2007; Lam *et al.*, 2016; Li *et al.*, 2017; Peng *et al.*, 2018; Guo *et al.*, 2019). Subject head motion and magnetic field drift that occurred during the MRSI scan were detected and corrected using navigator signals acquired simultaneously. The frequency shifts induced by magnetic field inhomogeneity and susceptibility effects were corrected using the high-resolution field map determined from the companion water signals. Spectral quantification was done using an improved LCmodel-based algorithm that incorporated both spatial and spectral priors (Li *et al.*, 2017). The estimated metabolite concentrations were normalized using the water reference to compensate for the B_1_ inhomogeneity of the head coil used in the experiment. A more detailed description of SPICE is provided in the Supplementary material.

### Arterial spin labelling perfusion-weighted MRI

To obtain the perfusion images in the initial scan, the pseudo-continuous arterial spin labelling (pCASL) PWI with multiple post-labelling delays was used (3.75 × 3.75 × 3.75 mm^3^, field of view = 240 mm, repetition time = 3.2 s, 3.4 s 3.9 s, 4.6 s, 5.4 s, echo time = 10.3 ms, inversion time = 150 ms, 34 slices, labelling duration = 1.5 s, post-labelling delays = 0.8 s, 1.0 s, 1.5 s, 2.2 s, 3.0 s). The ASL images were processed using the ASLtoolbox (Wang *et al.*, 2008) and the Statistical Parametric Mapping brain imaging analysis software (SPM12). The processing pipeline included: (i) realignment of the ASL images to the M0 reference images for head motion correction, spatially smoothing using a 3D isotropic Gaussian kernel with a full-width at half-maximum of 5 mm; and (ii) generation of the cerebral blood flow images using a two-compartment perfusion model. Anatomical grey matter, white matter and CSF masks were generated from the corresponding MPRAGE images using SPM12’s unified normalization/segmentation routine. The perfusion deficit area was defined by a threshold of 20 ml/100 g/min on the cerebral blood flow map within the grey matter mask (Harston *et al.*, 2015).

### Lesion and tissue segmentation

To delineate the acute DWI lesion, a threshold (620 × 10^−6^ mm^2^/s) was applied to the ADC data (Harston *et al.*, 2015), yielding a binary mask. See Supplementary Fig. 3 for an individual-level validation of the thresholds for DWI lesion and perfusion deficit area delineation. The final infarct area was manually defined on the follow-up FLAIR images. The masks of the DWI lesion, final infarct and perfusion deficit were all inspected by an experienced neuroradiologist and manually corrected where necessary, blinded to the appearance of the corresponding metabolic images. The following regions of interest were defined for tissue level analysis: (i) infarct core: tissue in both the acute DWI lesion area and the final FLAIR infarct area; (ii) infarct growth: tissue in the final FLAIR infarct area but not in the acute DWI lesion area; (iii) oligemia: tissue in the perfusion deficit area but not in the DWI lesion area and the final infarct area; and (iv) DWI reversal: tissue in the DWI lesion area but not in the final FLAIR infarct area. The definitions of masks are illustrated in Supplementary Fig. 1.

### Image registration

Image registration was done using FMRIB’s Linear Image Registration Tool and FMRIB’s Nonlinear Image Registration Tool (Jenkinson *et al.*, 2012). The FLAIR images, the ADC maps, the cerebral blood flow images and the corresponding tissue masks were all coregistered to the MRSI images (metabolic maps) using an affine transformation with 12 degrees of freedom (Jenkinson *et al.*, 2012). Contralateral non-ischaemic masks were created by transforming the DWI lesion masks to standard MNI152 space, flipping along the midline, and transforming back to the native image space. All the registration results were inspected for accuracy by an experienced neuroradiologist: no substantive inaccuracies were identified.

### Statistical analysis

We performed tissue-wise analysis for group comparisons using SPSS 24 (IBM). The metabolite concentrations were calculated relative to the sum of contralateral NAA, creatine and choline signals for tissue-wise analysis. Paired *t*-tests were used to compare the means between different regions of interest across patients. Pearson’s correlation analyses were performed to assess the strength of the linear relationship between metabolic signals and time from stroke onset. We used Bonferroni correction for multiple comparisons. For MRSI data, individual metabolites for which the Cramér–Rao lower bound lower than 20% were excluded from the regional analysis. This initial proof-of-concept study has no prior data from which a power analysis could be derived, and is not scaled to permit sensitivity analysis.

### Data availability

The data that support the findings of this study are available from the corresponding author, upon reasonable request.

## Results

**Figure 2:**
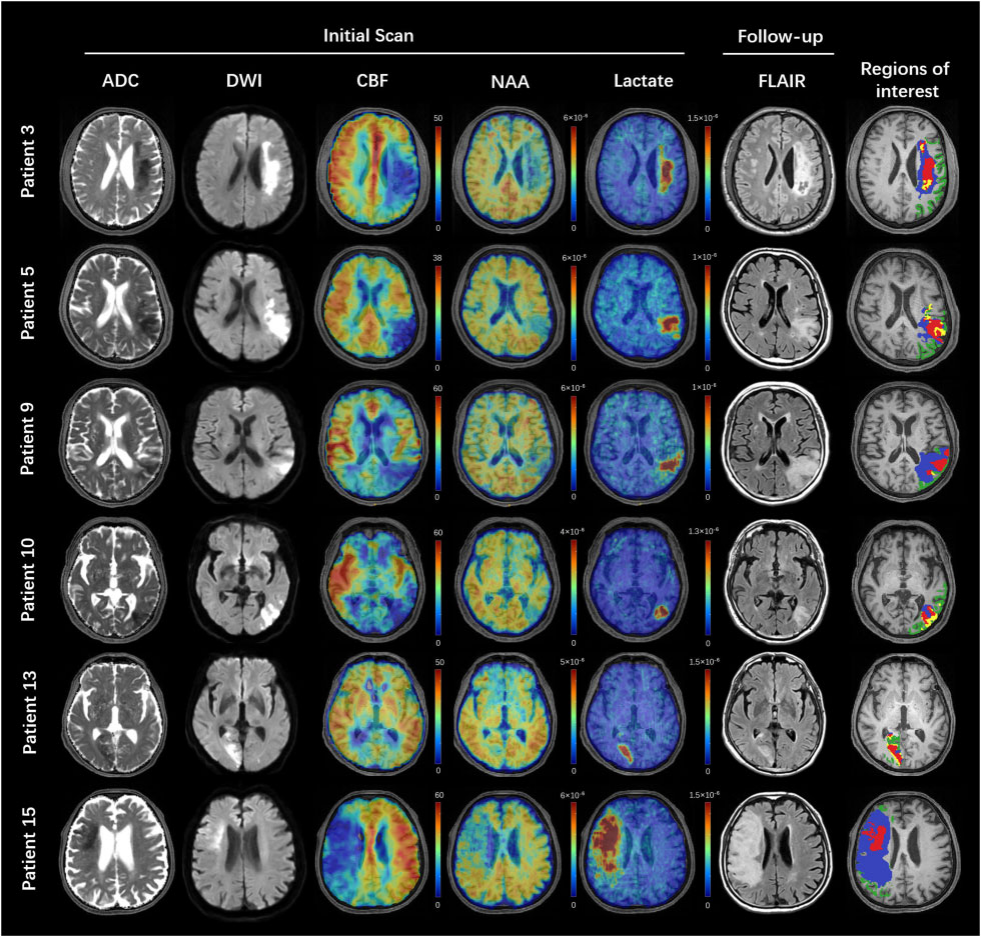
**Multimodal images from representative patients.** All the images were registered to the structural T_1_-weighted images. The ADC, DWI, cerebral blood flow and MRSI images were acquired in the first scan. The FLAIR images were acquired in the follow-up session. The colour bar for ASL-PWI shows the cerebral blood flow in ml/100 g/min. The colour bar for MRSI shows NAA or lactate level in institutional units. Regions of interest: light green = oligemia; blue = infarct growth; yellow = DWI reversal; red = infarct core.

Representative triplanar high-resolution 3D MRSI maps of lactate and NAA are shown in Fig. 1A and B, respectively. The spectra associated with voxels from the infarct core, the infarct growth area and the oligemic area are also illustrated. Reduction of NAA and increase in lactate are readily apparent in the infarct core compared with other regions. Multimodal images including ADC, DWI, ASL-PWI, MRSI and FLAIR maps from six representative patients are shown in Fig. 2 and Supplementary Fig. 2. Inspection of the metabolic images shows obvious differences in signal in the vicinity of the lesion, especially with lactate.

**Figure 3:**
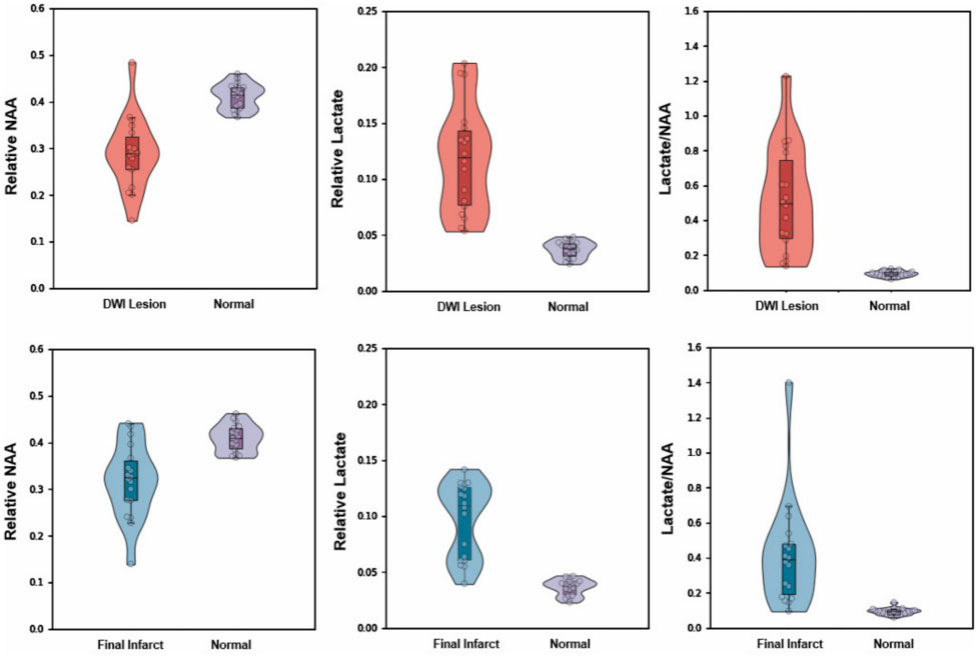
**Comparisons of mean relative NAA, lactate, and lactate/NAA between DWI lesion versus normal tissue (*top*) and between final infarct versus normal tissue (*bottom*).** Boxes indicate the interval between 25th and 75th percentiles; horizontal lines indicate median values; whiskers indicate the interval between 1.5 times the interquartile range above the 75th percentile and the corresponding distance to the 25th percentile value. Violin plots show the distribution of the data using kernel density estimation with automatic bandwidth selection.

We focused on NAA and lactate in our analyses for their plausible sensitivity to the metabolic consequences of ischaemia. Formal statistical comparisons of the metabolite signal within the DWI lesion versus contralateral normal tissue, and separately within the final infarct versus contralateral normal tissue, establish the significance of the observed differences across the cohort (Fig. 3 and Supplementary Figs 5 and 6). In each case, both NAA (*P < *0.0001) and lactate (*P < *0.0001) were significantly different. The ratio of the two amplified the effect in size (*P < *0.0001). The threshold of significance was α  <  0.0056 (0.05/9) after correction for multiple comparisons. Analysis of the data using water signal as the reference revealed similar results (Supplementary Fig. 9).

**Figure 4:**
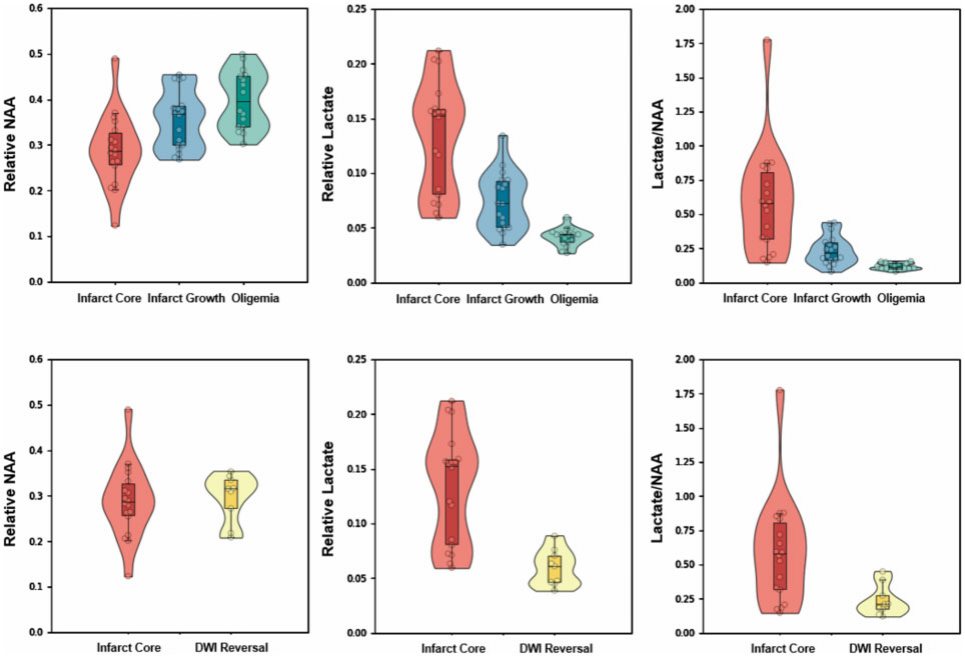
**Comparisons of mean relative NAA, lactate, and lactate/NAA among different regions within hypoperfused tissue (*top*) and DWI lesion (*bottom*).** Boxes indicate the interval between 25th and 75th percentiles; horizontal lines indicate median values; whiskers indicate the interval between 1.5 times the interquartile range above the 75th percentile and the corresponding distance to the 25th percentile value. Violin plots show the distribution of the data using kernel density estimation with automatic bandwidth selection.

A comparison of metabolites within hypoperfused tissue and the DWI lesion are shown in Fig. 4 and Supplementary Figs 4, 7 and 8. The infarct core exhibited lower NAA than the infarct growth area (*P < *0.001). The lactate signal within the infarct core was significantly higher than that within the infarct growth area (*P < *0.0001), which in turn was higher than that in the oligemia region (*P < *0.0001). The ratio of lactate to NAA further enhanced the observed regional differences. Within the DWI lesion, the DWI reversal area showed a significantly lower lactate signal (*P < *0.001) than that in the infarct core. The level of significance was α  <  0.0042 (0.05/12) after correction for multiple comparisons. Analysis of the data using water signal as the reference revealed similar results (Supplementary Fig. 10). No significant correlations were found for the metabolic signals within any of the tissue volumes with time from stroke onset (Supplementary Table 2).

## Discussion

We have demonstrated the feasibility and clinical potential of high-resolution, near whole-brain, 3D MRSI in acute stroke. Although the first clinical study of MRSI in stroke, conducted more than 25 years ago, stimulated significant interest in incorporating metabolic information in the assessment of brain tissue viability after stroke, technical limitations have constrained the state-of-the-art to large, single-voxel or 2D imaging, with partial coverage, and often clinically impracticable acquisition time (Barker *et al.*, 1994; Gillard *et al.*, 1996; Wild *et al.*, 2000; Nicoli *et al.*, 2003; Munoz Maniega *et al.*, 2008; Cvoro *et al.*, 2009). We have succeeded in scaling metabolic imaging to high-resolution, full coverage, 3D volume while reducing the data acquisition time to well within the clinically acceptable range. We obtained 3D maps of NAA and lactate concurrently at a nominal spatial resolution of 2.0 × 3.0 × 3.0 mm^3^ in acute stroke patients in 8 min. Key to the success of SPICE in this application are: (i) built-in capability for water referencing; (ii) motion correction; (iii) detection and correction of magnetic field inhomogeneity and drift; and (iv) denoising capability. These features made our study possible and may prove useful for wider stroke imaging applications.

Here we analysed only the NAA and lactate signals within the multiple metabolites that SPICE is able to map at high resolution across the brain. Lactate directly reflects anaerobic glycolysis and tissue acidosis, which is closely associated with final tissue outcome in both experimental and clinical studies (Munoz Maniega *et al.*, 2008; Cvoro *et al.*, 2009; Lei *et al.*, 2009). Reliable extraction of lactate is challenging with traditional spectroscopic imaging methods owing to low signal-to-noise ratio and severe signal contamination from subcutaneous lipids and macromolecules (Dani *et al.*, 2012). Here these challenges were successfully overcome with the use of high-resolution acquisition and the advanced data processing methods in SPICE (Ma *et al.*, 2016; Guo *et al.*, 2019); the macromolecular signals were also successfully separated from the lactate signal via a subspace-based approach, exploiting their different spectral characteristics.

Our results revealed, for the first time, that the lactate signal could separate benign oligemia from the infarct growth region within the area of perfusion-diffusion mismatch. This finding reflects the observations based on 2D pH-weighted imaging (Harston *et al.*, 2015). Preserved NAA, along with an elevated lactate level, has long been hypothesized to represent a metabolic signature of penumbral tissue in previous studies (Gillard *et al.*, 1996; Nicoli *et al.*, 2003; Dani *et al.*, 2012; Dani and Warach, 2014). Munoz Maniega *et al.* (2008) found higher NAA values in ‘possibly abnormal’ voxels compared with the ‘definitely abnormal’ voxels in ischaemic lesions, whereas lactate was high in both. Dani *et al.* (2012) showed preserved NAA and elevated lactate within the PWI-DWI mismatch region. However, the use of single slice, 2D imaging, voxel at >10 mm, has obstructed the development of a clinically useful sequence. That 3D high-resolution MRSI could differentiate the PWI-DWI mismatch area into acidotic penumbra (high lactate and reserved NAA) and benign oligemia (low lactate and non-detectable NAA loss) could have significant clinical implications.

The metabolic heterogeneity of DWI lesions has been previously demonstrated with both PET and 2D-MRSI (Nicoli *et al.*, 2003; Guadagno *et al.*, 2006). The observed high incidence of ADC reversal (6.7% to 50%) shows that DWI abnormalities need not always represent irreversibly damaged tissue (Inoue *et al.*, 2014). This limits the value of the ADC map for predicting infarct size. Nicoli *et al.* (2003) found that a 33% decrease of mean ADC values within the DWI lesion was associated with a 122% increase of the lactate/NAA ratio. Our study clearly demonstrates the variations in lactate production within the area of restricted diffusion. Crucially, lactate differed between the infarct core and DWI reversal area (Fig. 4). Our results show that the metabolic biomarkers provided by SPICE significantly correlate with tissue viability.

While our study demonstrates the feasibility of 3D metabolic imaging of acute stroke within a real-world clinical setting and its potential benefits, further improvements of the technology could improve its clinical applicability. First, it is desirable to reduce the time needed for magnetic field shimming before the start of the MRSI scan. Manual shimming in our study took ∼3–4 mins, which could be reduced to less than 1 min with fast, automatic shimming methods. Second, SPICE data processing and reconstruction were performed off-line, using non-optimized code, taking ∼3 h on a relatively modest workstation (24 cores, 48 threads, 2.6 GHz, 256 GB memory). Since all the data processing algorithms are parallelizable, dramatic acceleration could be achieved with a relatively inexpensive multi-core supercomputing system, especially by taking advantage of graphics processing unit capability. Finally, the correction of motion/frequency drift was performed in post-processing using the navigator signals acquired during the MRSI scan. Sequence robustness could be further enhanced by performing the correction in real time, facilitating potential clinical applications.

Clinical heterogeneity is one of the challenges of stroke imaging. This pilot study evaluated an unselected cohort of patients presenting with acute stroke to a specialist stroke unit, which enabled us to test the robustness of our technique within a range of real-world symptom onset time and lesion characteristics. It is encouraging that despite the wide variation in clinical presentations, we could detect consistently the degradation of NAA and the increase of lactate in acute stroke, demonstrating the real-world feasibility and applicability of 3D high-resolution MRSI. Larger studies will be needed to quantify the variability of the metabolic biomarkers across the full range of clinical presentations, including variations in stroke symptom onset time and lesion characteristics. For example, a future study could specifically address the hyperacute stage stroke metabolic features, which is of great clinical relevance for thrombolytic interventions, and where the rapidity of lactate accumulation in ischaemia (Rehncrona *et al.*, 1981; Cvoro *et al.*, 2009) could yield a robust early signal of tissue threat.

In conclusion, high-resolution 3D MRSI has the potential to address a currently unmet need for non-invasive metabolic imaging in acute stroke (Kidwell *et al.*, 2003; van Leyen *et al.*, 2019). Metabolic assessment enabled by MRSI may improve the delineation of ischaemic penumbra, and also provide additional insights in gaining a better understanding of regional vulnerability. Our feasibility study warrants further investigation of fast, high-resolution MRSI scan in stroke patients, in combination with other established imaging methods such as diffusion and perfusion weighted imaging.

## Funding

This study was supported by National Natural Science Foundation of China (81871083 and 61671292), and Shanghai Jiao Tong University Scientific and Technological Innovation Funds (2019QYA12). P.N. is funded by the Wellcome Trust (213038/Z/18/Z) and the UCLH NIHR Biomedical Research Centre.

## Competing interests

The authors report no competing interests.

## Supplementary material

Supplementary material is available at *Brain* online.

## Supplementary Material

awaa264_Supplementary_Data

## Abbreviations

ADC =: apparent diffusion coefficient
ASL =: arterial spin labelling
DWI =: diffusion weighted imaging
MRSI =: magnetic resonance spectroscopic imaging
NAA =: *N*-acetylaspartate
PWI =: perfusion weighted imaging
SPICE =: spectroscopic imaging by exploiting spatiospectral correlation
