# Supplementary material for: Fast high-resolution metabolic imaging of acute stroke with 3D magnetic resonance spectroscopy
Source: Brain. 2020 Nov 3;143(11):3225–33. doi: 10.1093/brain/awaa264 (PMC7719019; doi:10.1093/brain/awaa264)
Supplement: awaa264_Supplementary_Data [file awaa264_supplementary_data.pdf]

Supplementary Table 1 - Patient Characteristics

Patient	Stroke syndrome	Hemisp here	Sex	Age	NIH SS	Thromb olysed	Onset to scan (hrs)	Follow-up MRI (days)
1	PACS	Right	M	59	7	N	6	34
2	PACS	Left	M	40	4	N	24	96
3	TACS	Left	M	49	7	N	14	30
4	PACS	Left	M	63	12	Y	3	12
5	PACS	Left	M	78	4	N	13	8
6	LACS	Left	F	82	1	N	15	29
7	LACS	Right	M	69	3	N	15	8
8	LACS	Left	M	67	4	N	6	10
9	PACS	Left	F	77	2	N	12	7
10	PACS	Left	M	77	7	N	24	9
11	PACS	Right	F	84	5	N	24	15
12	PACS	Right	M	62	12	N	12	9
13	POCS	Right	M	68	9	N	8	7
14	PACS	Right	F	78	7	N	22	10
15	PACS	Right	M	81	10	Y	2	9
16	PACS	Left	M	64	9	N	24	12
17	PACS	Left	M	51	4	N	12	7
18	PACS	Left	M	55	2	N	24	8

NIHSS = National Institute for Health Stroke Scale; LACS = lacunar stroke; TACS = total anterior circulation stroke; PACS = partial anterior circulation stroke; POCS = posterior circulation stroke.

Supplementary Figure 1

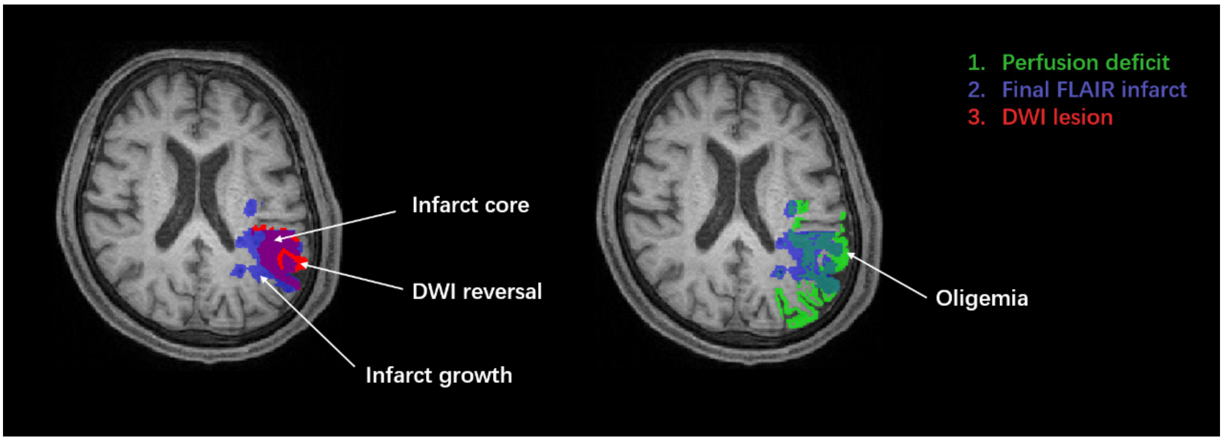

Figure 1. Regions of interest used in our study. Infarct core is the tissue in both the acute DWI lesion area and the final FLAIR infarct area. Infarct growth is the tissue in the final FLAIR infarct area but not in the acute DWI lesion area. Oligemia is the tissue in the perfusion deficit area but not in the DWI lesion area and the final infarct area. DWI reversal is the tissue in the DWI lesion area but not in the final FLAIR infarct area.

Supplementary Figure 2

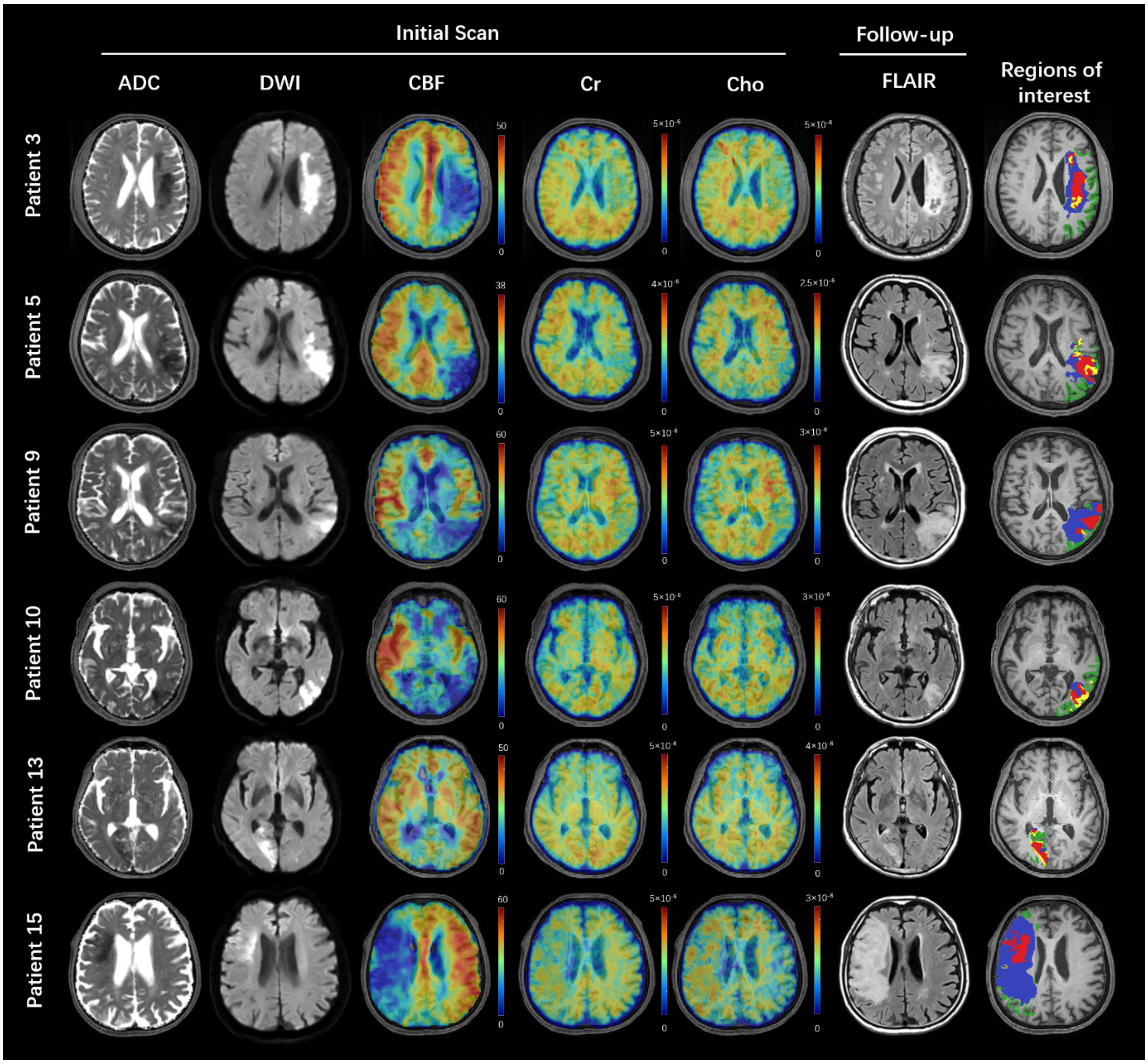

Figure 2. Creatine and choline maps from representative patients. The color bar shows creatine or choline level in institutional units.

Supplementary Figure 3

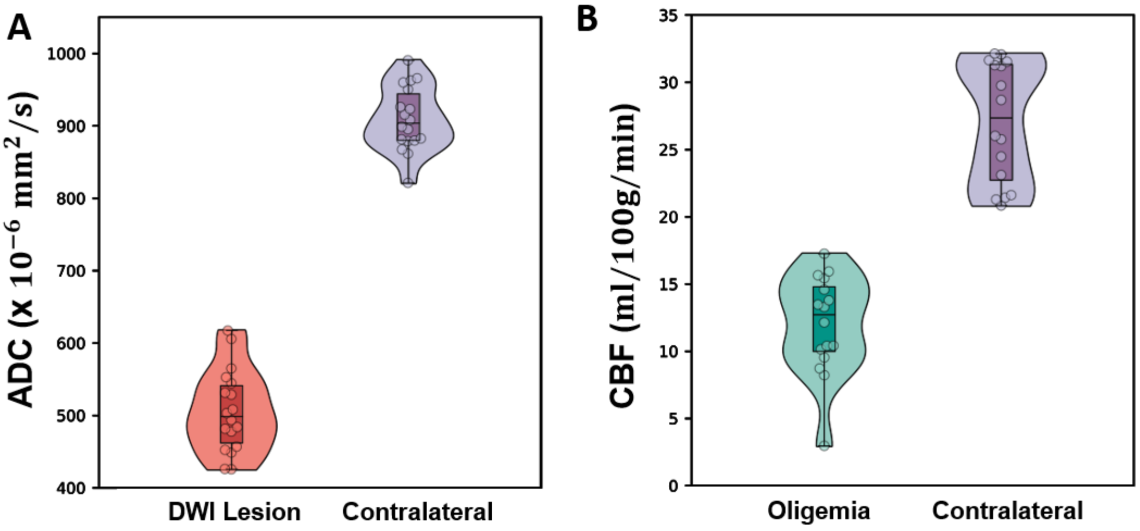

Figure 3. (A) Patient-level mean ADC for DWI lesion and contralateral hemisphere. (B) Patient-level mean CBF in oligemia and contralateral hemisphere.

Supplementary Table 2 – Correlations of Metabolic Signals with Time from Stroke Onset

Metabolic Signal / Region	**Relative NAA**	**Relative Lactate**	**Lactate/NAA**
Infarct Core	r = -0.4327<br>p = 0.0729	r = -0.0491<br>p = 0.8467	r = 0.3353<br>p = 0.1737
Infarct Growth	r = -0.0121<br>p = 0.9619	r = -0.3529<br>p = 0.1509	r = -0.3020<br>p = 0.2232
Oligemia	r = -0.3248<br>p = 0.2197	r = -0.3739<br>p = 0.1537	r = -0.1312<br>p = 0.6282
DWI Reversal	r = -0.1949<br>p = 0.6153	r = 0.2007<br>p = 0.6046	r = 0.2946<br>p = 0.4415
DWI Lesion	r = -0.3929<br>p = 0.1068	r = -0.0887<br>p = 0.7265	r = 0.2838<br>p = 0.2537
Final Infarct	r = -0.3592<br>p = 0.1432	r = 0.01878<br>p = 0.9410	r = 0.3830<br>p = 0.1167
Normal Tissue	r = -0.0937<br>p = 0.7116	r = -0.1398<br>p = 0.5800	r = -0.2033<br>p = 0.4185

Pearson correlation analysis was performed to measure the strength of the linear relationship between metabolic signals and time from stroke onset (in hrs).

Supplementary Figure 4

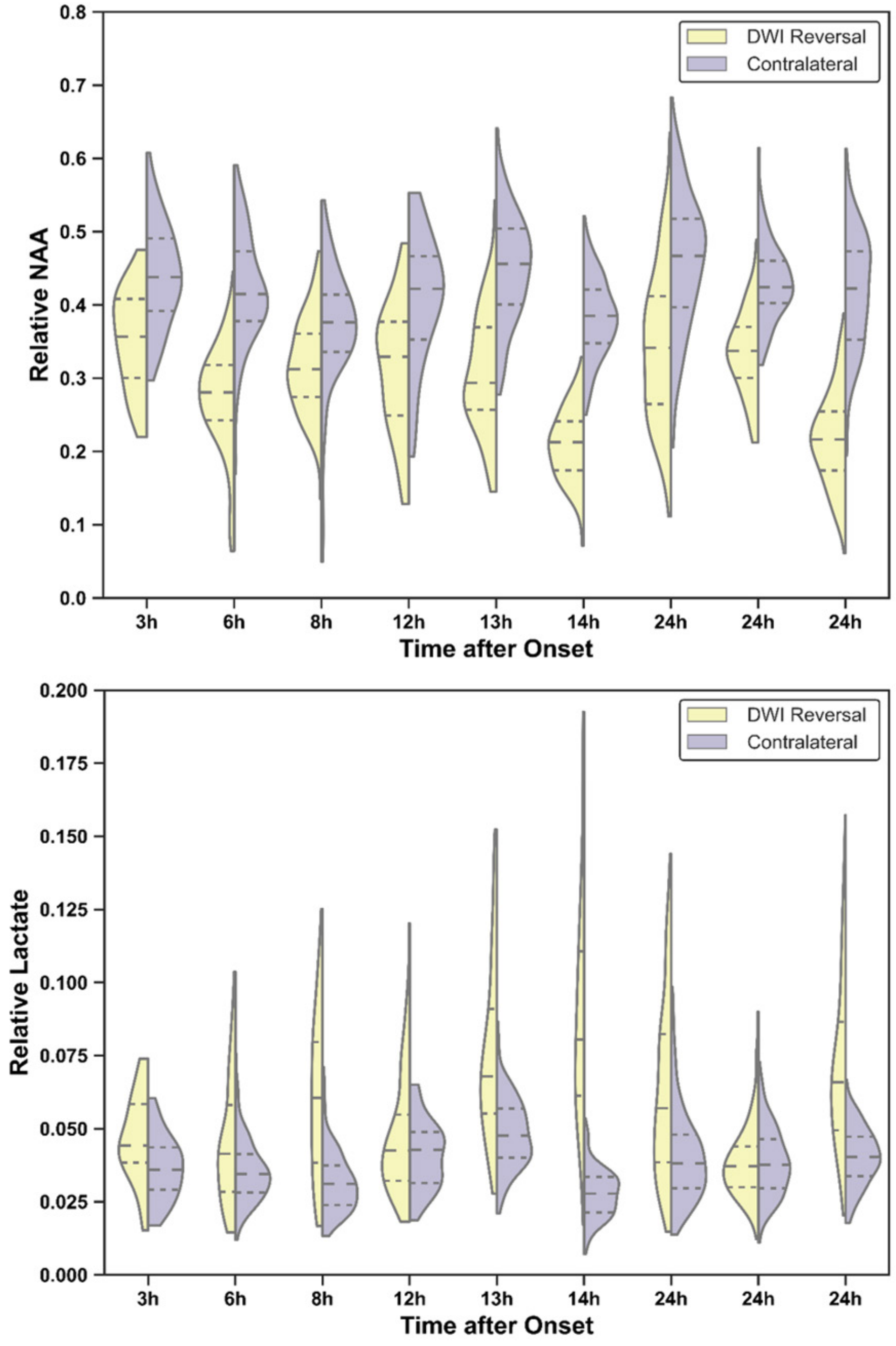

Figure 4. Metabolic signal distributions within the DWI reversal region in comparison with its contralateral compartment for each individual subject in our study, ordered by time from symptom onset. Note that only nine patients had DWI reversal regions. Split violin plots show kernel density estimates of the distributions of the metabolic signal for each subject within the lesion compartment (left side) and within the anatomically homologous voxels on the contralateral side (right side). Dashed lines indicate the median, dotted lines the 25th and 75th quartile intervals respectively.

Supplementary Figure 5

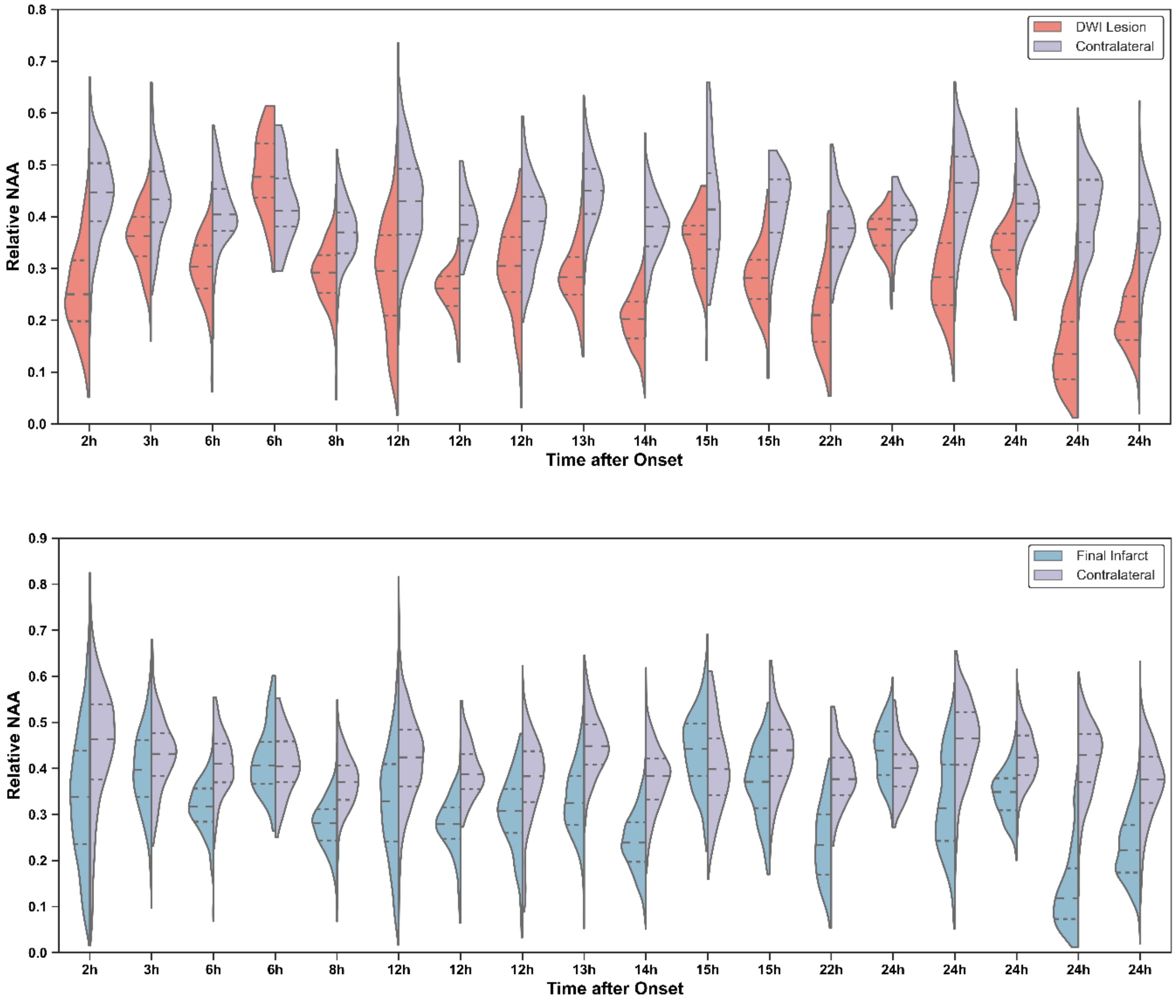

Figure 5. NAA signal distributions within DWI lesion (top) and final infarct (bottom) in comparison with their contralateral compartment, respectively, for each individual subject in our study, ordered by time from symptom onset. Split violin plots show kernel density estimates of the distributions of the metabolic signal for each subject within the lesion compartment (left side) and within the anatomically homologous voxels on the contralateral side (right side). Dashed lines indicate the median, dotted lines the 25th and 75th quartile intervals respectively.

Supplementary Figure 6

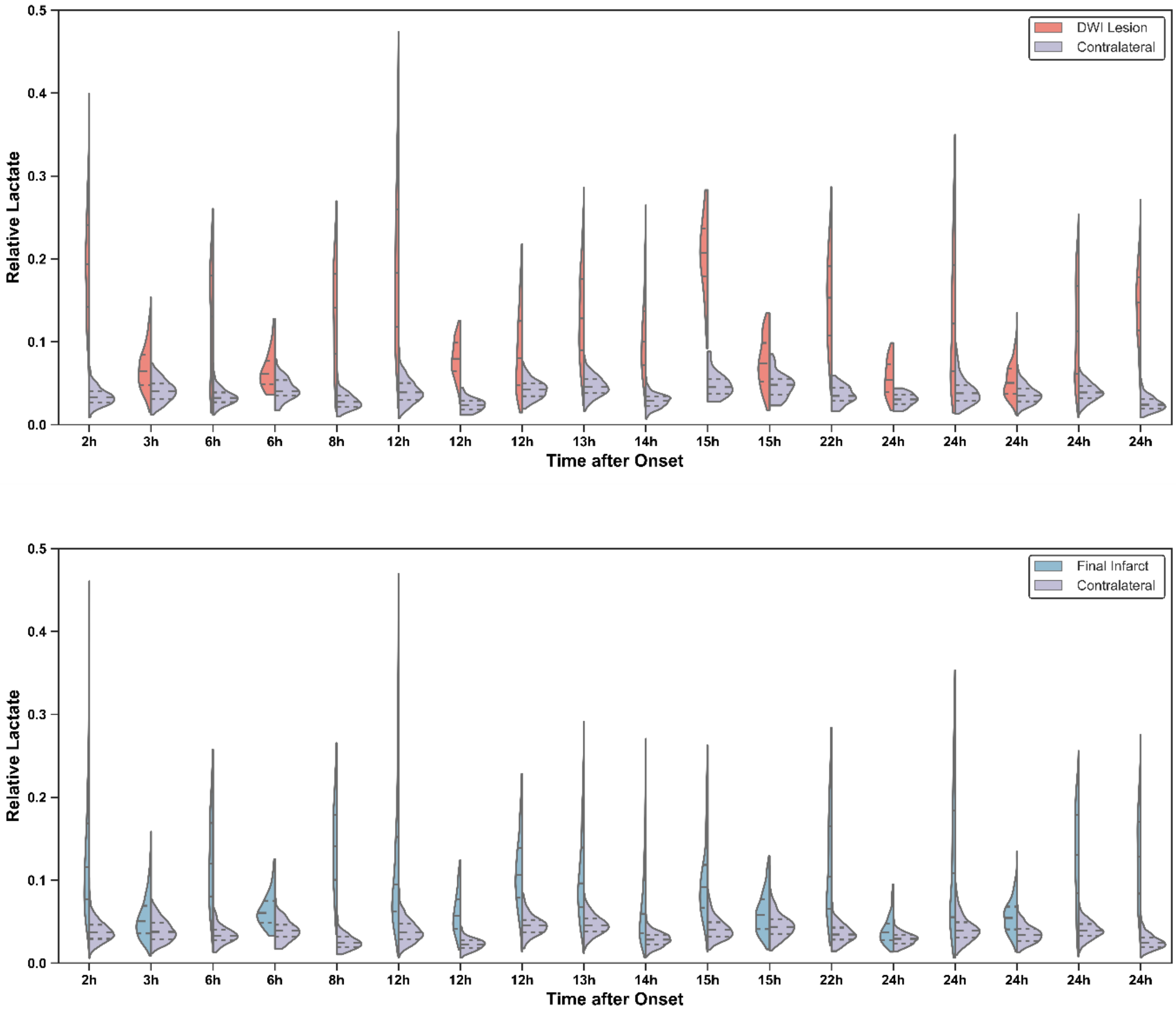

Figure 6. Lactate signal distributions within DWI lesion (top) and final infarct (bottom) in comparison with their contralateral compartment, respectively, for each individual subject in our study, ordered by time from symptom onset. Split violin plots show kernel density estimates of the distributions of the metabolic signal for each subject within the lesion compartment (left side) and within the anatomically homologous voxels on the contralateral side (right side). Dashed lines indicate the median, dotted lines the 25th and 75th quartile intervals respectively.

Supplementary Figure 7

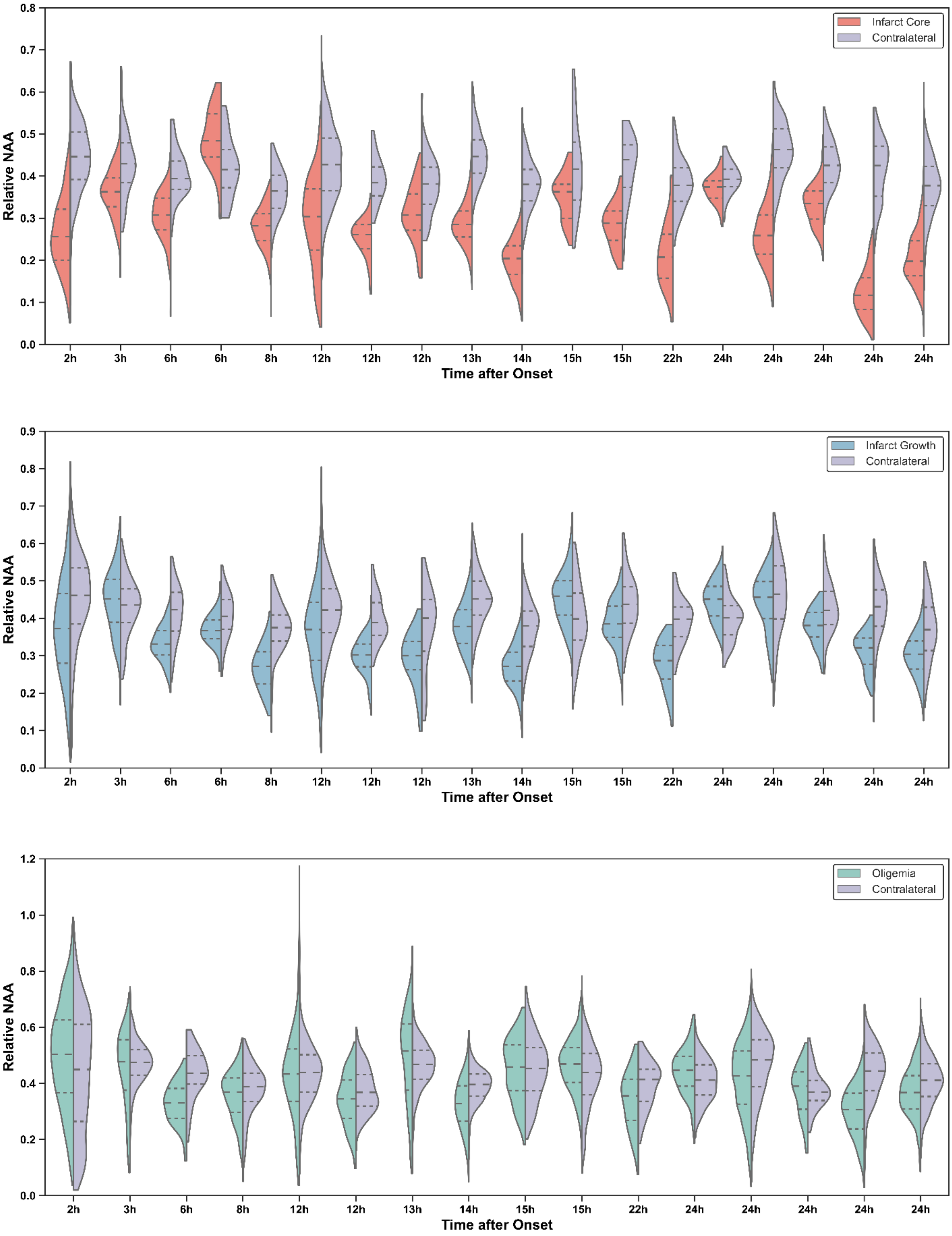

Figure 7. NAA signal distribution within each hypoperfused tissue in comparison with its contralateral compartment for each individual subject in our study, ordered by time from symptom onset. Split violin plots show kernel density estimates of the distributions of the

metabolic signal for each subject within the lesion compartment (left side) and within the anatomically homologous voxels on the contralateral side (right side). Dashed lines indicate the median, dotted lines the 25th and 75th quartile intervals respectively.

Supplementary Figure 8

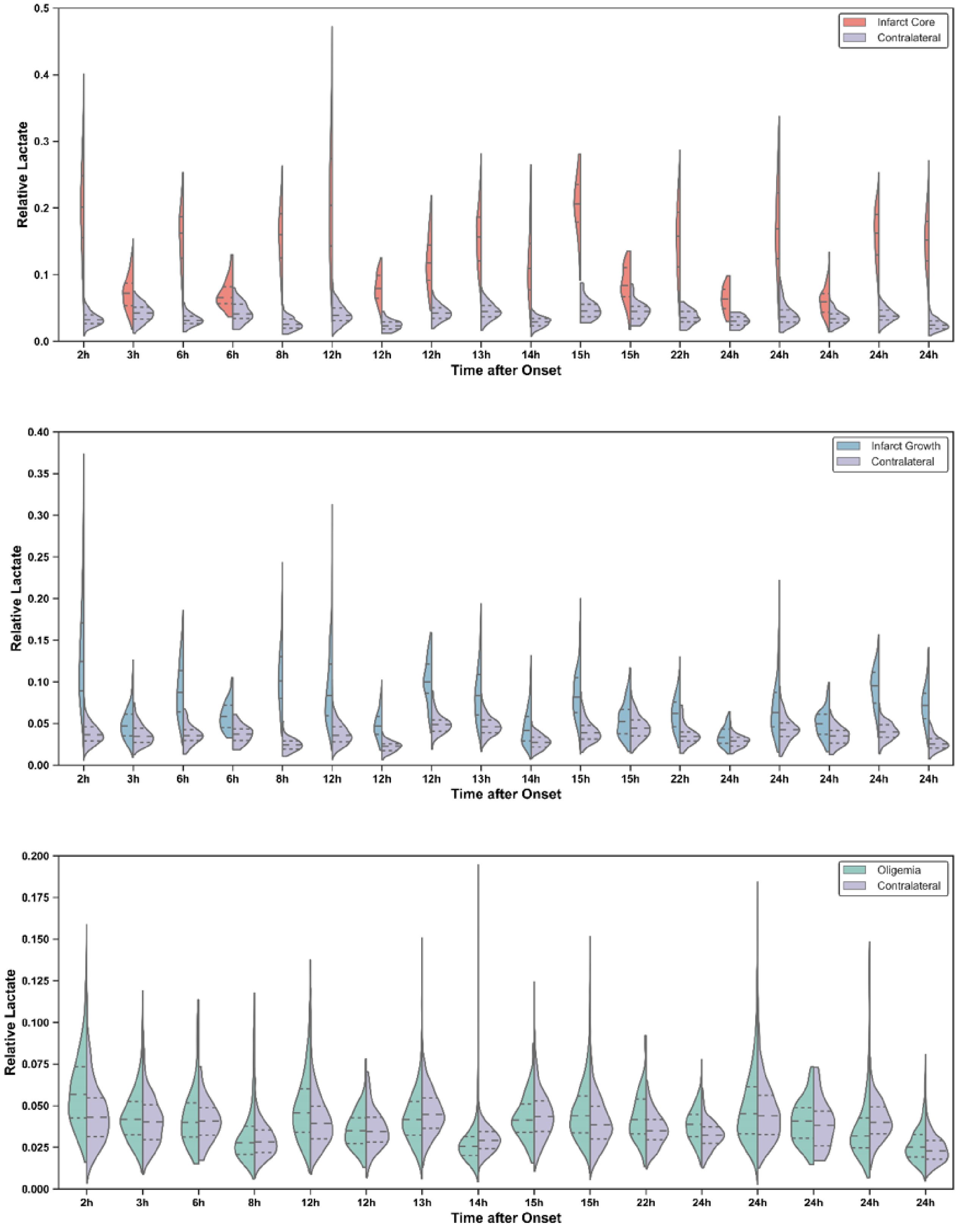

Figure 8. Lactate signal distribution within each hypoperfused tissue in comparison with its contralateral compartment for each individual subject in our study, ordered by time from

symptom onset. Split violin plots show kernel density estimates of the distributions of the metabolic signal for each subject within the lesion compartment (left side) and within the anatomically homologous voxels on the contralateral side (right side). Dashed lines indicate the median, dotted lines the 25th and 75th quartile intervals respectively.

Supplementary Figure 9

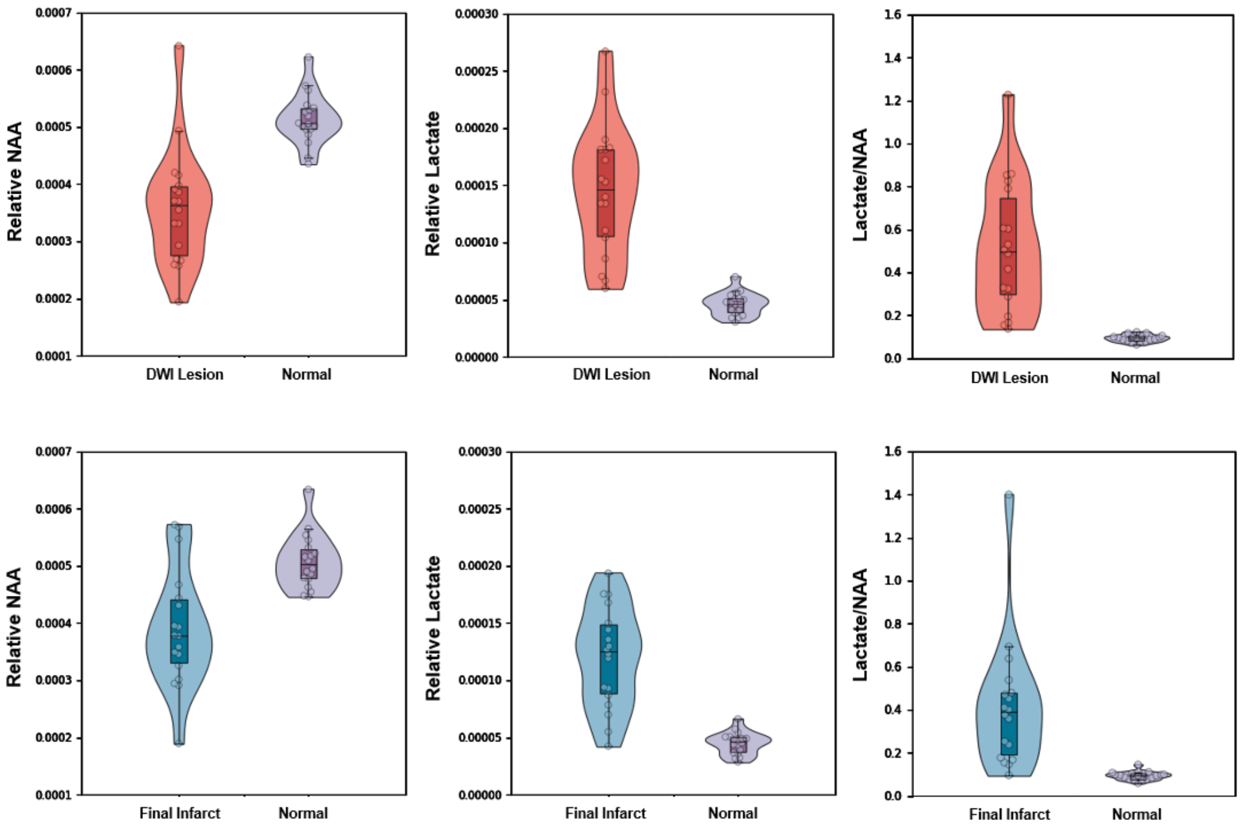

Figure 9. Comparisons of mean relative NAA, lactate and lactate/NAA between DWI lesion vs normal tissue (top) and between final infarct vs normal tissue (bottom). The normalization was performed using water signal as the reference. Boxes indicate the interval between 25th and 75th percentiles; horizontal lines indicate median values; whiskers indicate the interval between 1.5 times the interquartile range above the 75th percentile and the corresponding distance to the 25th percentile value. Violin plots show the distribution of the data using kernel density estimation with automatic bandwidth selection. Note: all p values <0.0001.

Supplementary Figure 10

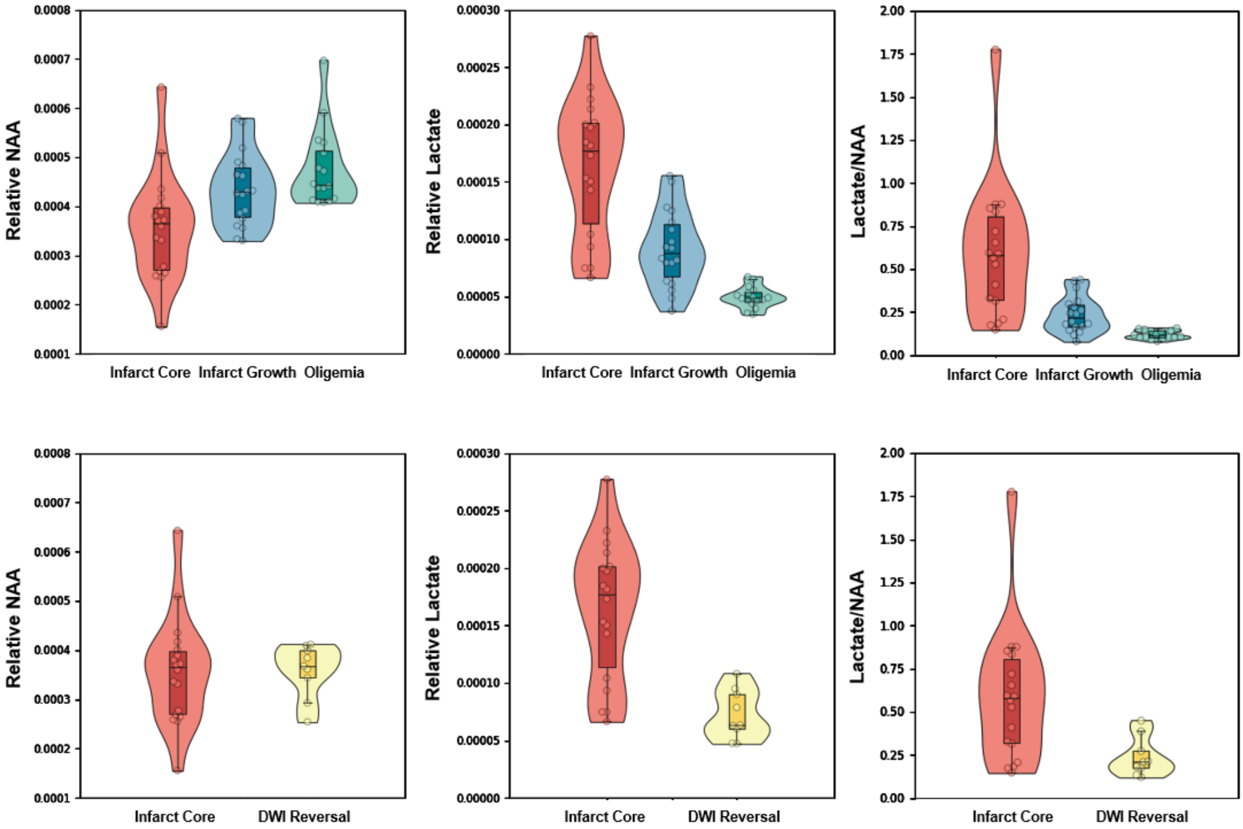

Figure 10. Comparisons of mean relative NAA, lactate and lactate/NAA among different regions within hypoperfused tissue (top) and DWI lesion (bottom). The normalization is performed using water signal as the reference. Boxes indicate the interval between 25th and 75th percentiles; horizontal lines indicate median values; whiskers indicate the interval between 1.5 times the interquartile range above the 75th percentile and the corresponding distance to the 25th percentile value. Violin plots show the distribution of the data using kernel density estimation with automatic bandwidth selection.

Supplementary Method

SPICE (SPectroscopic Imaging by exploiting spatiospectral CorrElation) is a subspace based MRSI technique, which exploits a unique property known as partial separability of spectroscopic signals. This property indicates that high-dimensional spectroscopic signals reside in a very low-dimensional subspace and enables special data acquisition and image reconstruction strategies to be used to obtain high-resolution spatiospectral distributions with good signal-to-noise ratios (SNR).

Data Acquisition

The pulse sequence used in our experiments is shown in Fig. 11. We included several novel features to enable fast, high-resolution, 3D metabolic imaging: a) elimination of the water and lipid suppression pulses used in traditional MRSI data acquisition methods, b) acquisition of free induction decays with ultrashort echo time (1.6 ms) and short TR, and c) sampling of the (k, t)-space in echo-planar spectroscopic imaging-based trajectory for rapid acquisition of spatiotemporal encodings. In our experiments, the central k-space was fully sampled to preserve the signal-to-noise ratio of the metabolite signals, while the peripheral (k, t)-space was sparsely sampled using blipped gradients to achieve high resolution for the water and lipid signals. To enhance robustness, we also acquired a set of navigator signals in 10 s interval for tracking B_0 field drift and object head motion. The sequence was implemented on a 3T MR scanner; the SPICE scans covered a field of view of 240 x 240 x 72 mm^3 in 8 mins with the following parameters: flip angle = 27°, echo time = 1.6 ms, repetition time = 160 ms, bandwidth = 167 kHz, matrix size of metabolite signals = 110 x 78 x 24 (corresponding to 2.0 x 3.0 x 3.0 mm^3 spatial resolution). *Because SPICE uses 3D volumetric acquisitions with high bandwidth RF pulses and large frequency encoding gradients, the influence of variable chemical shifts of different molecules on localization is minimal. More specifically, in our experiments, the transmit RF-bandwidth was around 8 kHz and the frequency encoding bandwidth per pixel was 1.51 kHz. The maximal chemical shift difference between metabolites was around 300 Hz at 3T. Therefore, its influence on slab-select excitation was around 3.8%, which was adequately handled with 20% oversampling in the slab-select direction; chemical shift displacement in the*

frequency encoding direction was about 0.2 pixel.

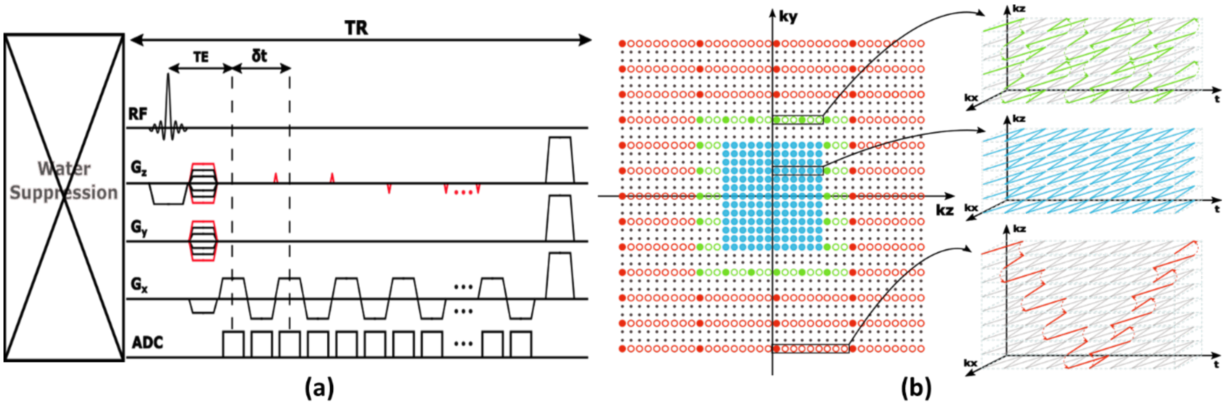

Figure 11: Illustration of the SPICE data acquisition sequence. (a) Sequence diagram showing the spatiospectral encoding scheme used; the gradients marked by red were used for blipped phase encodings to cover the peripheral k-space. (b) Coverage of (k, t)-space: the central segment of k-space is fully sampled; the medial segment is under-sampled by a factor of 3 in both ky and t; the outer segment is under-sampled by a factor of 3 in ky and a factor of 9 in t.

Data Processing

SPICE uses a union-of-subspaces mathematical framework for data processing. More specifically, it models the measured spectroscopic imaging signals as (1-4):

$$\rho(\boldsymbol{r},t) = \sum_{l_w=1}^{L_w} U_{l_w}(\boldsymbol{r})V_{l_w}(t) + \sum_{l_f=1}^{L_f} U_{l_f}(\boldsymbol{r})V_{l_f}(t) + \sum_{l_m=1}^{L_m} U_{l_m}(\boldsymbol{r})V_{l_m}(t),$$

where $\{V_{l_x}\}$ and $\{U_{l_x}\}$ are the spectral basis functions and spatial coefficients, and the subscript $x \in \{w, f, m\}$ specifies water, lipid and metabolite signals, respectively. This model reduces the degrees-of-freedom for representing the spatiospectral distribution of each molecule and allows effective separation of different molecular components based on their distinct spectral basis functions (1-6).

The key data processing steps in SPICE are summarized below:

(a) **Subspace learning:** The subspace structures for the water and lipid signals were learned directly from the imaging data (6), while the spectral bases for the metabolites were learned from training data exploiting their known resonance structures determined from quantum simulation (5).

(b) **Nuisance signal removal:** The unsuppressed water and lipids signals were removed from the measured SPICE data using a model-based method incorporating the water/lipid spectral priors (7). After water/lipids removal, the residual water sidebands were further removed using the method proposed in (8).

(c) **Reconstruction of spatiospectral functions:** The desired spatiospectral function of each molecule was determined by solving the following constrained reconstruction problem (1-4):

$$\min_{\{U_{l_m}\}} \left\| d - \Omega_k \mathcal{F}\left(\sum_{l_m=1}^{L_m} U_{l_m}(\boldsymbol{r}) V_{l_m}(t)\right) \right\|_2^2 + R(U_{l_m})$$

where d denotes the water/lipids removed data, $\mathcal{F}$ the imaging operator, Ω_k the sampling operator and $R(\cdot)$ the edge-preserving regularization functional.

(d) **Spectral Quantification:** Spectral quantification of the reconstructed metabolite signals was done using an improved LCmodel-based method, exploiting the union-of-subspaces modelling and spectral priors (5). The quantified concentrations were normalized to the companion water references for subsequent data analysis.

References

1. Lam F, Liang ZP. A subspace approach to high-resolution spectroscopic imaging. Magn Reson Med 2014; 71(4): 1349-57.
2. Lam F, Ma C, Clifford B, Johnson CL, Liang ZP. High-resolution ^{1}H-MRSI of the brain using SPICE: Data acquisition and image reconstruction. Magn Reson Med 2016; 76(4): 1059-70.
3. Peng X, Lam F, Li Y, Clifford B, Liang ZP. Simultaneous QSM and metabolic imaging of the brain using SPICE. Magn Reson Med 2018; 79(1): 13-21.
4. Guo R, Zhao Y, Li Y, Li Y, Liang ZP. Simultaneous metabolic and functional imaging of the brain using SPICE. Magn Reson Med 2019; 82(6): 1993-2002.
5. Li Y, Lam F, Clifford B, Liang ZP. A Subspace Approach to Spectral Quantification for MR Spectroscopic Imaging. IEEE Trans Biomed Eng 2017; 64(10): 2486-9.

6. Lam F, Li Y, Guo R, Clifford B, Liang ZP. Ultrafast magnetic resonance spectroscopic imaging using SPICE with learned subspaces. Magn Reson Med 2020; 83: 377-390.
7. Ma C, Lam F, Johnson CL, Liang ZP. Removal of nuisance signals from limited and sparse ^{1}H-MRSI data using a union-of-subspaces model. Magn Reson Med 2016; 75(2): 488-97.
8. Li Y, Lam F, Guo R, Clifford B, Peng X, Liang ZP. Removal of water sidebands from ^{1}H-MRSI data acquired without water suppression. Proc. Intl. Soc. Magn Reson Med 2018; p. 4016.
